# Supplementary material for: A Validated IVRT Method to Assess Topical Creams Containing Metronidazole Using a Novel Approach
Source: Pharmaceutics. 2020 Feb 3;12(2):119. doi: 10.3390/pharmaceutics12020119 (PMC7076423; doi:10.3390/pharmaceutics12020119)
Supplement: Supplementary file 1 [file pharmaceutics-12-00119-s001.pdf]

pharmaceutics

Supplementary Materials: A Validated IVRT Method to Assess Topical Creams Containing Metronidazole Using a Novel Approach

Seepraranі Rath and Isadore Kanfer

The following tables contain the release rates using six VDCs for each of the products.

A] Raw data for the results presented in Table 5 of the manuscript:

Table S1. 0.375% MTZ cream vs 0.75% MTZ cream.

Product	Release Rates (μg/cm²/min$^{1/2}$)					
	VDC 1	VDC 2	VDC 3	VDC 4	VDC 5	VDC 6
0.375% MTZ cream (Test)	13.838	16.712	16.012	18.616	15.872	16.435
0.75% MTZ cream (Reference)	34.147	34.583	30.514	33.433	34.472	36.440

Table S2. 1.125% MTZ cream vs 0.75% MTZ cream

Product	Release Rates (μg/cm²/min$^{1/2}$)					
	VDC 1	VDC 2	VDC 3	VDC 4	VDC 5	VDC 6
1.125% MTZ cream (Test)	56.250	55.176	51.200	52.870	56.207	52.579
0.75% MTZ cream (Reference)	34.147	34.583	30.514	33.433	34.472	36.440

Table S3. Metrocreme®, 0.75% MTZ (run 1) vs Metrocreme®, 0.75% MTZ (run 2).

Product	Release Rates (μg/cm²/min$^{1/2}$)					
	VDC 1	VDC 2	VDC 3	VDC 4	VDC 5	VDC 6
Metrocreme®, 0.75% MTZ (run 1) (Test)	39.508	38.792	37.362	37.429	34.850	39.255
Metrocreme®, 0.75% MTZ (run 2) (Reference)	39.784	39.983	39.128	38.756	38.704	39.405

Table S4. Metrocreme®, 0.75% MTZ (run 1) vs Metrocreme®, 0.75% MTZ (run 3).

Product	Release Rates (μg/cm²/min$^{1/2}$)					
	VDC 1	VDC 2	VDC 3	VDC 4	VDC 5	VDC 6
Metrocreme®, 0.75% MTZ (run 1) (Test)	39.508	38.792	37.362	37.429	34.850	39.255
Metrocreme®, 0.75% MTZ (run 3) (Reference)	40.452	39.873	38.984	41.647	44.411	39.878

Table S5. Metrocreme®, 0.75% MTZ (run 2) vs Metrocreme®, 0.75% MTZ (run 3).

Product	Release Rates (µg/cm²/min$^{1/2}$)					
	VDC 1	VDC 2	VDC 3	VDC 4	VDC 5	VDC 6
Metrocreme®, 0.75% MTZ (run 2) (Test)	39.784	39.983	39.128	38.756	38.704	39.405
Metrocreme®, 0.75% MTZ (run 3) (Reference)	40.452	39.873	38.984	41.647	44.411	39.878

B] Raw data for the results described on lines 337-338 of the manuscript for comparative IVRT:

Table S6. 0.75% MTZ cream vs Metrocreme®, 0.75% MTZ

Product	Release Rates (µg/cm²/min$^{1/2}$)					
	VDC 1	VDC 2	VDC 3	VDC 4	VDC 5	VDC 6
0.75% MTZ Cream (Test)	32.054	31.511	32.973	32.681	35.987	32.160
Metrocreme®, 0.75% MTZ (Reference)	38.558	40.085	35.820	36.989	35.467	39.880

C] Raw data for the results presented in Table 6 of the manuscript:

Table S7. Metrocreme®, 0.75% MTZ vs Metrocreme®, 0.75% MTZ

Product	Release Rates (µg/cm²/min$^{1/2}$)					
	VDC 1	VDC 2	VDC 3	VDC 4	VDC 5	VDC 6
Metrocreme®, 0.75% MTZ (run 1) (Test)	39.508	38.792	37.362	37.429	34.850	39.255
Metrocreme®, 0.75% MTZ (run 2) (Reference)	39.784	39.983	39.128	38.756	38.704	39.405

Table S8. 0.563% MTZ cream vs Metrocreme®, 0.75% MTZ

Product	Release Rates (µg/cm²/min$^{1/2}$)					
	VDC 1	VDC 2	VDC 3	VDC 4	VDC 5	VDC 6
0.563% MTZ cream (Test)	28.301	25.874	27.382	28.655	26.976	27.342
0.75% MTZ cream (Reference)	37.131	40.592	37.906	37.455	38.135	39.624

Table S9. 0.945% MTZ cream vs Metrocreme®, 0.75% MTZ

Product	Release Rates (μg/cm²/min$^{1/2}$)					
	**VDC 1**	**VDC 2**	**VDC 3**	**VDC 4**	**VDC 5**	**VDC 6**
0.563% MTZ cream (Test)	50.237	51.976	51.544	51.407	50.862	51.172
0.75% MTZ cream (Reference)	37.131	40.592	37.906	37.455	38.135	39.624

The statistical analyses were performed as follows:

The "sameness" between the two runs was assessed using the Mann- Whitney statistical test, which requires computation of a 90% confidence interval (CI). The first run was considered to be the reference while the second run was assigned to be the test. The test/reference (T/R) ratios of the calculated release rates (slopes) were computed, wherein R corresponds to the respective slopes of the first run and T corresponds to the release rates of the second run. A total of 36 T/R ratios were calculated from the combination of 6 Ts and 6 Rs. The 90% CI was determined by ordering these ratios from lowest to highest and extracting the 8th and 29th T/R ratios which were converted into percentages to become the lower and upper limits of the 90% CI respectively. The "sameness" between the two PVT runs can be established if the calculated 90% CI lies within the limits of 75 – 133.33% in accordance with the SUPAC-SS guidance.

© 2019 by the authors. Submitted for possible open access publication under the terms and conditions of the Creative Commons Attribution (CC BY) license (http://creativecommons.org/licenses/by/4.0/).
